# Hybrid Cellulose-Glass Fiber Composites for Automotive Applications

**DOI:** 10.3390/ma12193189

**Published:** 2019-09-28

**Authors:** Cindu Annandarajah, Amy Langhorst, Alper Kiziltas, David Grewell, Deborah Mielewski, Reza Montazami

**Affiliations:** 1 Department of Agricultural and Biosystems Engineering, Iowa State University, Ames, IA 50011, USA; cindu@iastate.edu; 2 Ford Motor Company, Research and Advanced Engineering, Dearborn, MI 48124, USAakizilt1@ford.com (A.K.); dmielews@ford.com (D.M.); 3 Department of Industrial and Manufacturing Engineering, North Dakota State University, Fargo, ND 58102, USA; david.grewell@ndsu.edu; 4 Department of Mechanical Engineering, Iowa State University, Ames, IA 50011, USA

**Keywords:** composites, hybrid fibers, cellulose, glass fiber, automotive, compatibilizer

## Abstract

In the recent years, automakers have been striving to improve the carbon footprint of their vehicles. Sustainable composites, consisting of natural fibers, and/or recycled polymers have been developed as a way to increase the “green content” and reduce the weight of a vehicle. In addition, recent studies have found that the introduction of synthetic fibers to a traditional fiber composite such as glass filled plastics, producing a composite with multiple fillers (hybrid fibers), can result in superior mechanical properties. The objective of this work was to investigate the effect of hybrid fibers on characterization and material properties of polyamide-6 (PA6)/polypropylene (PP) blends. Cellulose and glass fibers were used as fillers and the mechanical, water absorption, and morphological properties of composites were evaluated. The addition of hybrid fibers increased the stiffness (tensile and flexural modulus) of the composites. Glass fibers reduced composite water absorption while the addition of cellulose fibers resulted in higher composite stiffness. The mechanical properties of glass and cellulose filled PA6/PP composites were optimized at loading levels of 15 wt% glass and 10 wt% cellulose, respectively.

## 1. Introduction

Increasing global industrialization has resulted in environmental deterioration, including land and air pollution, leading to more global environmental awareness and promoting the investigation of environmentally friendly and sustainable materials. In addition, new legislation in large industrial markets, such as the European Union, has driven the automotive industry to prioritize global sustainability. Even though there is no federal law governing extended producer responsibility (EPR) in the United States, “product stewardship” practices call for shared responsibility among manufacturers and consumers to reduce product impact on the environment [1].

Currently, about 50% of the volume of materials in the cars are made of polymeric materials. The average usage of plastics in automotive in developed countries and globally averages are 120 kg and 105 kg, respectively, accounting for 10–12% of the total weight of a vehicle [2,3,4]. The Corporate Average Fuel Economy (CAFE) estimates that a reduction of 10% of an automobile’s weight will reduce its fuel consumption by 6–8% [3]. In attempts to lightweight and decrease the carbon footprint of vehicles, automakers have expressed increased interest in bio-based materials, especially natural-fiber reinforced polymer composites including kenaf, hemp, sisal, jute, and flax [5,6]. Automobile manufacturers have incorporated these natural fibers as the reinforcing phase for polymer composites in door panels, seat backs, headliners, package trays, dashboards, and interior parts. Use of these materials not only increases the “green content” of each vehicle, but can also contribute to reduction in weight, cost, carbon footprint, and lead to less dependence on foreign and domestic petroleum-based fuels [2,5]. These fiber based composites have the advantage to meet diverse design requirements with a 30% weight reduction and a cost reduction of 20% [7].

Development of hybrid composite reinforced with more than two types of fibers in matrix provides a more favorable balance of material properties. In hybridization, the formulation is made so that the fibers are able to support the loads while the matrix adheres the fiber together for efficient load transfer in the composite [8]. The properties of a hybrid composite are dependent on several factors, such as the nature of matrix; the nature, length, and relative composition of the reinforcements, fiber–matrix interface, and hybrid design [9]. For instance, Kalaprasad et al. studied the hybrid composited of sisal/glass reinforced polyethylene (SGRP) and reported that the mechanical properties were increased with increase in volume fraction of glass fibers [10]. In another study, Langhorst et al. found that increased fiber loading level in agave fiber filled polypropylene composite enhanced the stiffness of the material [11]. Joseph et al. investigated the mechanical properties and water sorption behavior of phenol–formaldehyde (PF) hybrid composites reinforced with banana fiber and glass fiber. It is found that the tensile, impact, and the flexural properties of the banana fiber-PF composites have been increased by the hybridization of glass fibers [12]. In another study, Kahl et al. reported that the glass fiber content helped in significant increase of the tensile strength and compared to cellulose fiber composites at an overall 16% vol. ratio [13].

The automotive industry has been looking into adapting polymer blends, such as the polypropylene (PP) and Nylon 6 (PA6) blends, for “under-the-hood” applications where thermal stability is a key parameter. PP is commonly used for automobile parts because it is inexpensive, highly processable, and exhibits high water/chemical resistance. However, the relatively low modulus and poor heat resistance of PP makes it unsuitable for use in under-the-hood components [14]. PA6, on the other hand, exhibits high heat resistance, tensile strength, modulus, resistance to corrosive chemicals, and has attractive surface appearance, but readily absorbs moisture resulting in dimensional instability. Thus, blends of PP and PA6 yield intermediate properties that can be suitable for engine covers, air intake manifolds, cooling, and heating components, and cylinder head cover [15,16]. On the contrary, the opposing polarities of PP and PA6 causes phase separation in the blend and could result in poor mechanical properties [16,17,18]. In order to improve the mechanical properties and the morphology of PP/PA6 blends, PP grafted maleic-acid anhydride (PPgMA) has been used as a reactive compatibilizer [17,19]. Many studies have investigated the use of polymers blends of PP/PA6 along with compatibilizing agents [20,21,22,23]. In addition, there are also works done on the use of short glass fiber as a filler or reinforcement in these polymer blends [24,25,26]. However, to the authors’ best recollection, there are no studies that have been published investigating the mechanical properties of natural fiber reinforced PA6/PP blends or even cellulose-glass fiber reinforced PP/ PA6 composites via injection molding, a technique that is used in the automotive industry. The main objective was to study the dispersion of cellulose and glass fibers in recycled PP/PA6 blends, and to examine the fiber hybridization effect on the mechanical, morphological, and water absorption properties of these polymer blends.

## 2. Materials and Methods

### 2.1. Procurement

Post-consumer recycled polyamide 6 (PA6) and polypropylene (PP) were supplied by Wellman Advanced Materials (Johnsonville, SC, USA). Maleic anhydride-*grafted*-polypropylene (PPgMA) with a grafting level of 0.5 wt% maleic-anhydride (Fusabond P613) was supplied by DuPont (Wilmington, DE, USA). Cellulose fiber (~150 µm × 20 µm × ~2 µm) and glass fiber (~6–10 mm) were obtained from International Paper (Memphis, TN, USA) and PPG Industries Inc. (Pittsburgh, PA, USA), respectively. The supplied PP and PA6 had melting points of approximately 160 °C and 220 °C, respectively.

### 2.2. Extrusion and Injection Molding

Nine samples were produced, and their formulations are shown in Table 1. Each formulation consisted of a 70:30 wt% ratio of PA6: PP and, 6 wt% PPgMA. All composites contained 15–30 wt% glass fibers and 10–30 wt% cellulose fibers. Control groups consisting of an unfilled PA6/PP blend, a 30% glass filled PA6/PP blend, and a 30% cellulose filled PA6/PP blend were produced.

Extrusion was completed using a twin-screw laboratory extruder (ThermoHaake Rheomex Model PTW25, Thermo Fisher Scientific, Waltham, MA, USA). Prior to extruding, the materials were dried for at least 12 hours at 60 °C in a conventional oven to reduce the moisture content to less than 1% in the fibers and the possibility of hydrolysis the nylon in the blend. The blend pellets and the hybrid fibers (cellulose and glass fiber) were separately starve-fed into the extruder using K-Tron gravimetric feeders (Coperion, Stuttgart, Germany), and the extruded samples were immediately immersed into a room-temperature water bath. Extruder temperatures ranged from 205 to 220 °C from the hopper to the die. The compounded materials were pelletized using a lab-scale chopper. Pellets were dried in a conventional oven for at least 12 hours at 60 °C to reduce moisture content less than 1% before being injection molded into test specimens using a Boy Machine model 80M injection molding machine (BOY, Exton, PA, USA). Molding occurred using barrel temperatures ranging from 175 to 185 °C, with a mold temperature of 26 °C.

## 3. Test Procedures

### 3.1. Mechanical Testing

Tensile, flexural, and impact tests were performed according to ASTM D638-10 (2010), ASTM D790-10 (2010), and ASTM D256-10 (2010), respectively. All samples were tested in an environmentally conditioned room maintained at 23 °C and 50% relative humidity. Tensile tests were performed on an Instron 3366 machine (Instron, Norwood, MA, USA) using a 5 kN load cell, 50 mm extensometer, and extension rate of 5 mm/min. Three-point bend flexural tests were performed using a 50 mm wide support span and were run at a crosshead speed of 1.25 mm/min. Notched Izod impact tests were performed using a TMI machine (model 43-02-03), (Testing Machines Inc., New Castle, DE, USA) fitted with a 2 lb pendulum. At least six tensile, five flexural, and ten impact test specimens were tested for each formulation.

The ultimate tensile strength, yield strength, elongation at break, and Young’s modulus were determined from the tensile stress-strain curves, while flexural strength and flexural modulus were determined from the flexural stress-strain curves. The impact strength was determined from the notched Izod impact tests.

### 3.2. Water Absorption Test

Water immersion tests were performed according to ASTM D570. At least six specimens of each formulation were pre-conditioned in a 50 °C oven for 24 h to remove all moisture. All the samples were cooled at room temperature for 30 min and then weighed to the nearest 0.001 g. The conditioned samples were then immersed completely in a container of distilled water at 23 °C. The samples were weighed again to the nearest 0.001 g after 24 h, seven days, 21 days, and 35 days until the increase in weight for 3 consecutive weighing averages less than 1%. 

### 3.3. Microscopy

The fractured surfaces of the tensile specimens were mounted vertically on sample holder to expose the composite’s cross-section perpendicular to the injection molding flow direction. The cross-sections of samples were observed using a FEI Quanta FEG 250 field emission SEM (Thermo Fisher Scientific, (Waltham, MA, USA). A 10 keV beam was used and backscatter electron images were collected. The low vacuum mode was used with 60 Pa water vapor environment. The working distance was set at approximately 10 mm.

### 3.4. Statistical Analysis

Results were analyzed by ANOVA using SAS (version 9.2; SAS Institute Inc., Cary, SC, USA) and XLStat (version 2013.4; Addinsoft, New York, NY, USA). A significance level of α = 0.05 with Tukey adjustment for multiple comparisons was used to determine significant differences.

## 4. Results and Discussion

### 4.1. Mechanical Properties

#### 4.1.1. Strength and Elongation 

**Figure 1:**
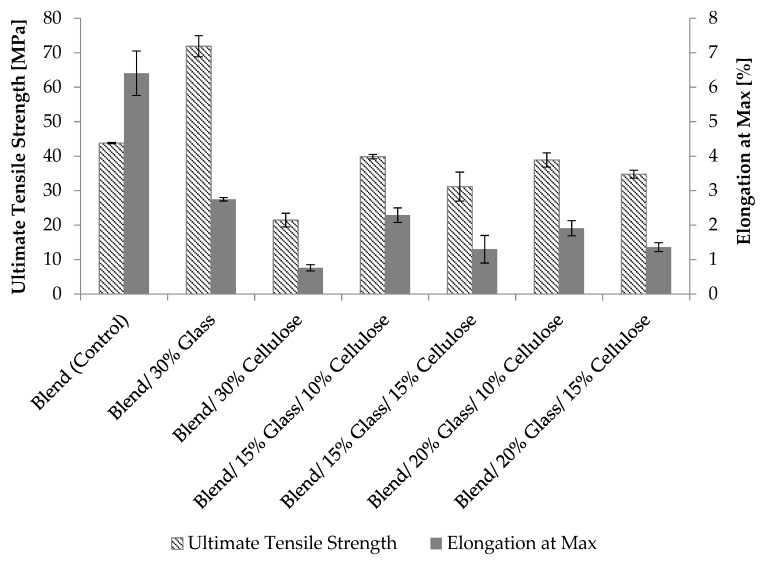
Tensile strength and elongation at maximum load of unfilled polymer blends and cellulose + glass fiber hybrid reinforced composites.

Figure 1 shows the tensile strength and strain at maximum load for the composites. The 30% glass fiber control sample exhibited the highest tensile strength (64% more than unfilled control) while the unfilled control exhibited the highest elongation at the ultimate strength. Contrary to cellulose being a high elongation fiber [6], the 30% cellulose control exhibited the lowest elongation at the ultimate strength. The significant reduction in both tensile strength and elongation may have been caused by agglomeration of cellulose fibers at high loading levels as well as poor adhesion between fiber and matrix. This result is in agreement with Sukri et al., who reported that extension was reduced by 63% when kenaf fiber loading levels were increased from 0 to 30% in rPP/rPA6 blends [17]. Santos et al. also reported that the tensile strength and extension of PA6 and Curaua fiber composites (with fiber content from 20 to 40%) was lower compared to the PA6 control sample [18].

No significant differences were observed in tensile strength and elongation of composites containing 15 or 20% glass fibers. It is speculated that the effect of the glass fiber reinforcement in these composites is maximized near 15% loading level. In more detail, strength as a function of glass content asymptotically approaches a maximum level around 15% filler levels. The increment of filler ratio could also cause more fiber ends to act as stress generators during deformation of composites and this results in causing earlier fiber-matrix debonding and crack development during deformation [27,28]. This assumption is supported by a study by Mishra et al. in which the tensile strength of sisal/glass hybrid polyester composites did not increase with the addition of glass fibers beyond 5.7 wt%. Similarly, the author reported that the tensile strength of a pineapple leaf/glass hybrid polyester composite decreased by about 10% when loaded with more than 12.9% glass fiber [9]. This result is also in line with the studies by Franciszczak et al. in which, addition of 14 vol%. glass fiber with PP gave merely 27% further increase in composite’s tensile strength [28]. In general, as the cellulose content in the composites increased, the tensile strength and elongation was reduced. While cellulose is a high elongation fiber [6], this reduction may be attributed to the decreased strength of cellulose compared to glass fiber. The glass fiber, relatively stiff, failed first which caused the transfer of the applied load to the cellulose fibers. The hybrid composite exhibited medium elongation (higher compared to the cellulose control but lower compared to the glass control) compared to the individual fiber reinforced composites [12].

**Figure 2:**
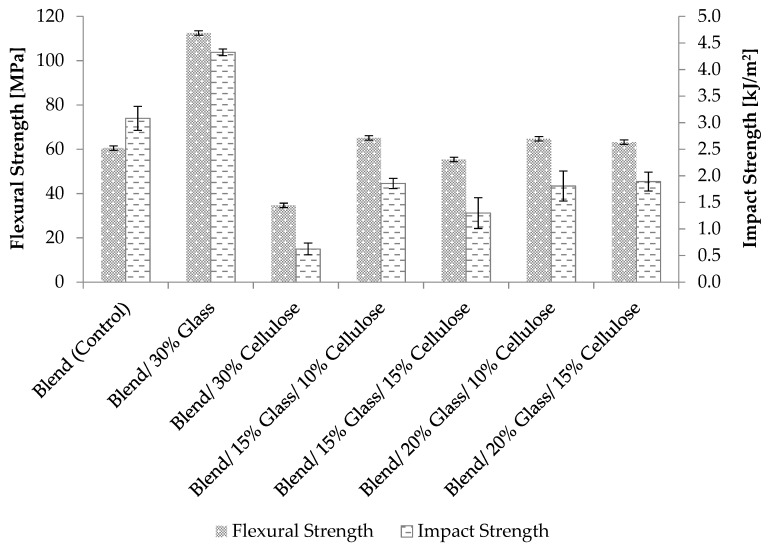
Flexural and impact strength of unfilled polymer blends and cellulose and glass fiber reinforced composites.

The results of flexural strength and impact strength for PP, PA6, PPgMA, cellulose, and glass fiber composites are shown in Figure 2. The 30% glass fiber control and 30% cellulose control samples exhibited the largest and smallest flexural and impact strengths, respectively. Results from composites containing 15% glass fiber suggest that increasing cellulose loading-levels reduced the flexural and impact strength of the hybrid composites. The impact strength of fiber-reinforced composites depends on many factors, including the nature of the constituents, fiber/matrix adhesion, construction, and geometry of the composites, and test conditions. It is possible that cellulose fiber agglomeration resulted in the formation of stress concentrations, reducing the energy needed to initiate crack propagation.

Additionally, replacement of cellulose fiber with glass fiber resulted in an increase in flexural and impact strength. For example, 15% glass + 15% cellulose composites (30% total fiber content) exhibited lower flexural and impact strength compared to 20% glass + 10% cellulose composites (30% total fiber content) (Figure 2). This is consistent with previous studies that reported an increase in impact strength with glass fiber content. For example, Misra et al. observed a 34% increase in impact strength by the addition of 8.5% glass fiber to sisal-fiber-reinforced polyester composites. In a three-point flexure test, failure occurred as a result of bending failure and shear failure [9]. It is possible that higher glass fiber content prevents shearing, resulting in an increase in flexural strength.

#### 4.1.2. Stiffness

**Figure 3:**
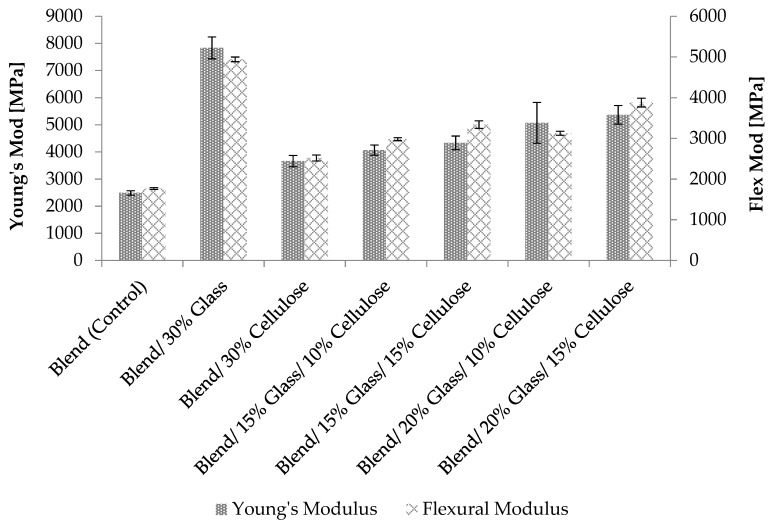
Young’s modulus and flexural modulus of unfilled polymer blends and cellulose and glass fiber reinforced composites.

Figure 3 shows the Young’s and flexural moduli for the composites. The 30% glass composites exhibited the highest moduli of all tested composites. It is also important to note that the hybrid composite of glass and cellulose fiber results in higher flexural strength than the tensile strengths implying the composite has better response to compression stress than tensile. In general, Young’s and flexural moduli increased with increasing cellulose and glass fiber content and these results are in agreement with the Rule of Hybrid Mixture (ROHM). This result is in agreement with Kahl et al., who reported the increase in tensile modulus with increasing content of glass fibers in the PP/glass fiber and cellulose composite due to the higher modulus of glass fiber [13]. Several mechanisms, such as compression, shearing *etc*., take place together during the stiffness test [29]. The significant increase in stiffness is due to the reinforcement effect of the cellulose and the increased resistance to shearing with the addition of glass fiber. This result is supported by a study of Sukri et al. who studied rPP, rPA6 and kenaf fiber by varying the fiber content from 10 to 30% [17]. Lei et al. also found a 50% increase in modulus by adding 30 wt% of bagasse to recycled high density polyethylene (rHDPE) [30]. 

### 4.2. Water Absorption

**Figure 4:**
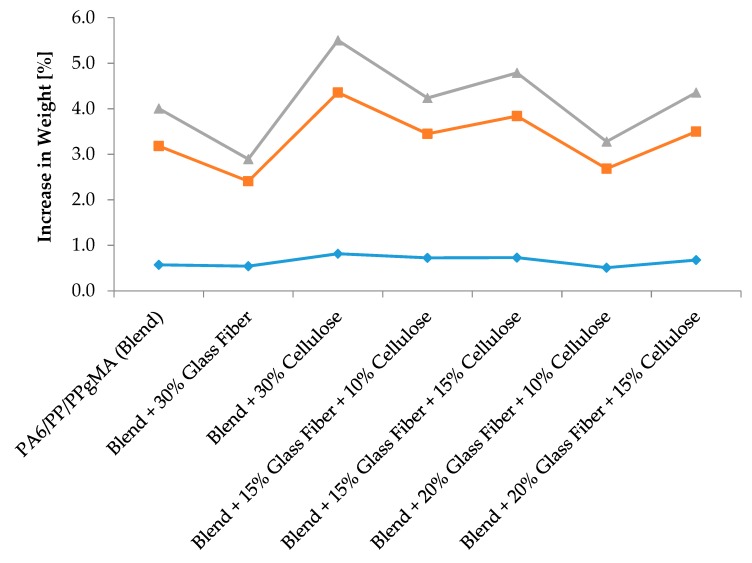
Water absorption of unfilled polymer blends and cellulose and glass fiber reinforced composites.

The water absorption of PA6/ PP/ PPgMA composites is shown in Figure 4. The highest and lowest water absorptions during a 3-week period was exhibited by 30% cellulose control (5.5%) and 30% glass fiber (2.89%), respectively. The hydrophilic OH groups on the surface of the cellulose crystallites or in the amorphous region may be available for bonding with water if there is no crosslinking with other OH groups. The water bonding at the amorphous region and the free water in the cellulose cavities cause an increase in absorption [31]. Among the composites, there was no significant difference in water absorption with increasing cellulose content. However, increasing the composite’s glass fiber content from 15 to 20% significantly reduced water absorption. The water enters through the interface and can diffuse through the porous structure of the fibers. Water penetration and diffusion mainly occur at the fiber–matrix interface and through the fibers via capillary mechanism [31]. When glass fiber was incorporated, the water uptake decreased, as the diffusion of water is not possible through glass fiber, as shown in Figure 4. In more detail, it is seen that in general, water absorption is inversely proportional to glass content. Similar observation has been seen by Joseph et al. who reported that the incorporation of a small amount of glass fiber (12%) increased the resistance of banana/PF composites to water sorption very effectively [12].

A summary of the mechanical results is shown in Table 2. Incorporation of glass fibers increased the strength and moduli of the PA6/PP/PPgMA blend composites. The cellulose control group reduced the strength, elongation, stiffness, and increased water absorption of the polymer composites. On average, the composites with glass/cellulose fiber mixtures showed only increased Young’s modulus and flexural modulus of the composite and a reduction in tensile strength and elongation compared to the unfilled control. It has no effect on the flexural, impact strength and water absorption (for 10 and 15% only). The addition of glass and cellulose fibers improved the stiffness of the polymer blend, but all composites showed lower strength and elongation compared to the unfilled polymer blend. In this study, 15% glass + 10% cellulose composites exhibited the best balance in properties. Sreekala et al. analyzed the tensile, elongation, and stiffness properties of phenol formaldehyde reinforced with glass and oil palm fibers composites as a function of fiber composition. The author reported that 40 wt% fiber loading, composites with 0.74 volume fraction of oil palm fiber (29.6% oil palm fiber, 10.4% glass fiber) exhibited the highest tensile properties among the hybrid composites as excellent dispersion of the fibers and increased oil palm fiber/glass compatibility occurs at this composition [29].

### 4.3. Morphological Properties

#### Scanning Electron Microscopy

**Figure 5:**
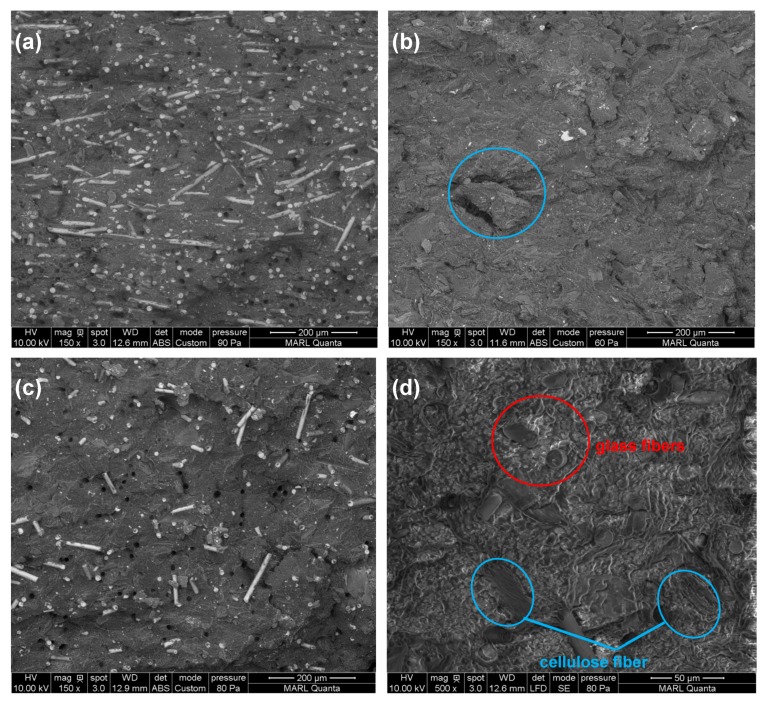
SEM images of the fracture surfaces of (**a**) blend + 30% glass fiber; (**b**) blend + 30% cellulose fiber (circled); and (**c**,**d**) blend + 15% glass fiber + 10% cellulose fiber at 150× and 500× respectively.

SEM images in Figure 5 show morphological differences between the control polymer blends and composite containing the blend and the glass and cellulose fibers. The micrograph of Figure 1a illustrates the well dispersion of glass fibers in the polymer blend matrix. Figure 1b shows cellulose fibers within a polymer blend matrix (cellulose fibers are circled). The matrix surrounding the cellulose fibers is cracked, suggesting that adhesion between the fiber and matrix is weak and this is in line with the low tensile strength achieved in the results. The micrographs of blend + 15% glass fiber + 10% cellulose fiber suggests that the area of the cellulose fibers is surrounded with glass fibers, which effectively reinforces the fiber and matrix together, leading to good interfacial adhesion between the two and this in result brings the tensile property of this formulation close to the one of neat blend formulation.

## 5. Conclusions 

This study determined the effects of adding cellulose and glass fibers on the mechanical, morphological, and water absorption properties of PP/PA6/PPgMA composites. In general, the mechanical properties were enhanced by addition of glass fibers alone: tensile strength, Young’s modulus, flexural modulus, flexural strength, and Izod impact strength were increased at 30% glass fiber load. Even though the composites with hybrid (cellulose and glass) fibers did not perform better than the control PP/ PA6 blend, increasing the cellulose content from 10 to 20% increased the Young’s and flexural modulus and water absorption, while flexural strength and elongation was reduced. In addition, when the glass fiber content was increased from 15 to 20% in the composite, the flexural strength, Young’s modulus and impact strength improved as well. There are no significant differences observed in the different loading levels from 10 to 30%. The 15% glass + 10% cellulose fiber composite showed the best properties among the composites with hybrid (cellulose and glass) fibers as the modulus and percentage elongation at break was the highest. Effect of fiber reinforcement on thermal properties and effect of compatibilizer should also be studied in the future to learn more about the thermal stability, crystallinity, compatibility, and to improve the properties of these composites.

## Figures and Tables

**Table 1:** Experimental design for the preparation of recycled polypropylene and Nylon 6 blends with hybrid fibers composites.

Formulation	Final Composition				
	PA6 (wt%)	PP (wt%)	PPgMA(wt%)	Glass (wt%)	Cellulose (wt%)
PA6/ PP/ PPgMA (Blend)	65.8	28.2	6	0	0
Blend + 30% Glass fiber	44.8	19.2	6	30	0
Blend + 30% Cellulose	44.8	19.2	6	0	30
Blend + 15% Glass fiber + 10% Cellulose	48.3	20.7	6	15	10
Blend + 15% Glass fiber + 15% Cellulose	44.8	19.2	6	15	15
Blend + 20% Glass fiber + 10% Cellulose	44.8	19.2	6	20	10
Blend + 20% Glass fiber + 15% Cellulose	41.3	17.7	6	20	15

**Table 2:** Details of mechanical properties for PP, PA6, and hybrid fibers (cellulose and glass fibers) composites. Samples containing the combination of one or both fibers were compared to their unfilled control, lacking the hybrid fibers. Boxes labelled (+) exhibited property improvement, white boxes with (o) experienced no significant property change, and boxes (-) experienced property degradation.

Properties		Single Filler Composites		Dual Filler Composites
		Glass Fiber	Cellulose	Glass Fiber + Cellulose
Strength	Tensile	+	-	-
	Flex	+	-	o
	Impact	+	-	o
Elongation	Tensile	-	-	-
Stiffness	Young’s Modulus	+	-	+
	Flexural Modulus	+	-	+
Absorptivity	Water Absorption	o	+	+
